# A radiomics approach for automated diagnosis of ovarian neoplasm malignancy in computed tomography

**DOI:** 10.1038/s41598-021-87775-x

**Published:** 2021-04-22

**Authors:** Shiyun Li, Jiaqi Liu, Yuanhuan Xiong, Peipei Pang, Pinggui Lei, Huachun Zou, Mei Zhang, Bing Fan, Puying Luo

**Affiliations:** 1 Department of Gynecology, Jiangxi Provincial People’s Hospital Affiliated to Nanchang University, Nanchang, 330006 China; 2 Department of Radiology, Jiangxi Provincial People’s Hospital Affiliated to Nanchang University, Nanchang, 330006 China; 3 GE Healthcare, Hangzhou, 310000 China; 4 Department of Radiology, The Affiliated Hospital of Guizhou Medical University, Guiyang, 550000 China

**Keywords:** Cancer, Cancer imaging, Oral diseases

## Abstract

This paper develops a two-dimensional (2D) radiomics approach with computed tomography (CT) to differentiate between benign and malignant ovarian neoplasms. A retrospective study was conducted from July 2017 to June 2019 for 134 patients with surgically-verified benign or malignant ovarian tumors. The patients were randomly divided in a ratio of 7:3 into two sets, namely a training set (of n = 95) and a test set (of n = 39). The ITK-SNAP software was used to delineate the regions of interest (ROI) associated with lesions of the largest diameters in plain CT image slices. Texture features were extracted by the Analysis Kit (AK) software. The training set was used to select the best features according to the maximum-relevance minimum-redundancy (mRMR) criterion, in addition to the algorithm of the least absolute shrinkage and selection operator (LASSO). Then, we employed a radiomics model for classification via multivariate logistic regression. Finally, we evaluated the overall performance of our method using the receiver operating characteristics (ROC), the DeLong test. and tested in an external validation test sample of patients of ovarian neoplasm. We created a radiomics prediction model from 14 selected features. The radiomic signature was found to be highly discriminative according to the area under the ROC curve (AUC) for both the training set (AUC = 0.88), and the test set (AUC = 0.87). The radiomics nomogram also demonstrated good calibration and differentiation for both the training (AUC = 0.95) and test (AUC = 0.96) samples. External validation tests gave a good performance in radiomic signature (AUC = 0.83) and radiomics nomogram (AUC = 0.95). The decision curve explicitly indicated the clinical usefulness of our nomogram method in the sense that it can influence major clinical events such as the ordering or abortion of other tests, treatments or invasive procedures. Our radiomics model based on plain CT images has a high diagnostic efficiency, which is helpful for the identification and prediction of benign and malignant ovarian neoplasms.

## Introduction

Ovarian tumors are common tumors of the female reproductive system. These tumors can be categorized into malignant or benign types, based on whether the tumor tends to become progressively worse (leading to deterioration or death) or not. Different types of ovarian tumors have different management and treatment schemes. Therefore, accurate identification of ovarian tumors as benign or malignant is highly crucial^1^.

Ovarian tumors are usually occult in the deep female pelvic cavity with insidious onset. The diagnosis of such tumors usually depends on the clinical experience of the gynecologists and the characteristics of the employed imaging technique, which might be ultrasonography, magnetic resonance imaging (MRI)^2^. Because of the subjectivity of ultrasonography, the expensive of MRI and the allergy prone of contrast agent, plain CT is optimal selection for this study on account of population usability and mass acceptance. However, diagnosis of the ovarian tumor malignancy has been traditionally based on the subjective qualitative judgment of radiologists and gynecologists who use their clinical experience to examine imaging data and assess ovarian tumors of high tissue diversity and heterogeneity^3^. Subjective evaluation is generally unstable under the influence of wide variations in the human rater expertise.

Radiomics is a new subfield of radiology that has recently emerged as an alternative to the traditional qualitative diagnosis approach^4,5^. In radiomics, imaging data quantification is assisted by a variety of advanced methods of image processing. In particular, algorithms for data characterization are utilized for deriving an immense number of numerical features from radiographic images^4,5^. Furthermore, numerous investigations have demonstrated that CT-based radiomics typically show high performance in the differentiation between benign and malignant lesions in several human organs including the kidneys, lungs, and liver^6^. Our work is based on the hypothesis that we can utilize CT-based radiomics features extracted from primary ovarian tumor lesions in order to establish imaging biomarkers that can non-invasively identify benign and malignant tumors, and also differentiate between them.

## Materials and methods

### General information

The current investigation is a retrospective one, which has been scrutinized closely and thoroughly, and then officially approved and accepted by the Ethics Committee of Jiangxi Provincial People’s Hospital Affiliated to Nanchang University. Informed consent was formally secured from all concerned parties, particularly patients. All the relevant guidelines and regulations that are agreed upon worldwide were observed while carrying out this work. We retrospectively reviewed relevant surgical and radiological data collected between 2017 and 2020. Persons selected for inclusion in this study satisfied the following criteria: (a) female patients with histopathological verified ovarian tumors, (b) persons with no history of previous or current malignancy other than that of ovarian tumors, (c) patients who were subjected to preoperative high-resolution procedures for ovarian cancer staging, and (d) patients who had preoperative CT for the pelvic area within the preceding half a month. Among patients satisfying these criteria, 38 patients were not included according to the following considerations: (1) patients who were subjected before the CT examination to radiotherapy, chemotherapy or chemoradiotherapy (n = 20). (2) patients diagnosed to suffer from inflammatory disease conditions (n = 11), (3) patients with low-quality imaging records (n = 7). We also selected 26 eligible patients from another hospital for external validation. Eventually, 160 patients were considered in our study.

### CT image acquisition

The CT images were obtained by the SOMATOM Definition CT scanner. We used automatic modulation with those scanning parameters: a tube voltage of 120 kVp, a tube current of 150 mAs, a section thickness of 5 mm, a reconstruction interval of 1 mm, and a slice gap of 1 mm.

### Region-of-interest segmentation

**Figure 1:**
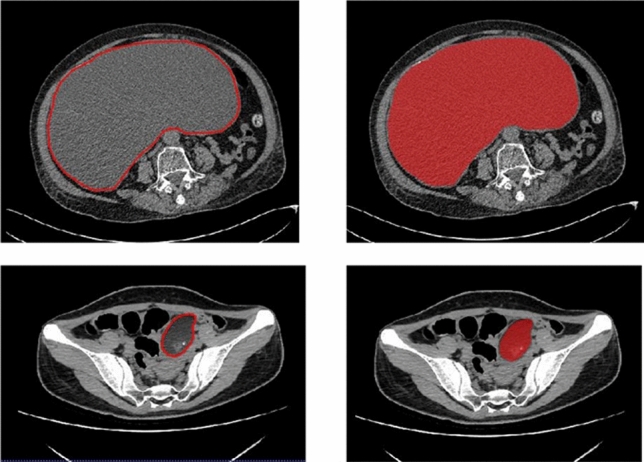
Manual delineation on the slice having the largest ovarian lesion diameter.

All regions of interest were segmented from baseline DICOM images using ITK-SNAP (Version 3.6.0). Manual ROI segmentation from the slice with the largest lesion diameter^7^ was performed independently by two radiologists (henceforth referred to as readers A and B, who have 5 and 15 years of abdominal radiology experience, respectively) (See Fig. 1).

### Feature extraction

We extracted textural features for 134 ROIs (62 Benign and 72 malignant) using the Artificial Intelligence Kit for life sciences (Version 3.0.1.A, GE Healthcare). For each ROI, a total of 396 features were computed including those of texture, histograms, form factors, gray-level co-occurrence matrices (GLCM), grey level run-length matrix (RLM), and gray-level zone-size matrices (GLZSM). GLCM and RLM in four directions (0°, 45°, 90°, 135°) and three displacements (1, 4, 7) were calculated to describe patterns or the spatial distribution of voxel intensities. The details are shown in the “Supplement S1”.

### Feature preprocessing

Before feature selection, three steps of feature preprocessing were performed: (1) replacing the outliers by the median of the same feature; (2) the control and patient groups are subdivided into training (n = 95) and test (n = 39) sets with an approximate ratio of 7:3; (3) Z-score data normalization is applied Z-score normalization was done in the training dataset to eliminate the differences in the value scales of extraction features. And both training and test datasets were normalized using the mean and standard deviation computed using in the training dataset alone. (In this normalization process, the mean value is subtracted from the original feature value and then the difference is divided by the standard deviation).

### Feature selection and model construction

The feature selection and model construction were performed in the training dataset. First, we tested the robustness and reproducibility of image features. Since the features were extracted based on the ROIs segmented by radiologists manually, we only used the features that were most robust against the manual segmentation among different radiologists^8^. The correlation coefficient for each feature was calculated between the feature set-1 (from Radiologist-A) and feature set-2 (from Radiologist-B) by using the Spearman rank correlation test. Features with correlation coefficients greater than 0.8 were regarded as robust features, since a correlation coefficient of 0.8 indicated a high consistency and repeatability^9^. Second, we employed the maximum-relevance minimum-redundancy (mRMR) algorithm to select the features by maximizing the correlation between selected features and differentiating benign and malignant, eliminating the redundancy between features. Next, the least absolute shrinkage and selection operator^10,11^ (LASSO) method was employed to further select the most useful features by penalty parameter tuning λ. We chose the optimal λ based on the minimum criteria according to tenfold cross-validation. The radiomics signature (Radscore) was then calculated for each case via a linear combination of selected features that were weighted by respective coefficients. 

### The radiomics nomogram construction and evaluation

Univariate logistic regression was exploited to find independent predictors for ovarian tumors. The candidate predictors included clinical factors (i.e., age, ascites, and boundary), biomarker expression (CA125)^12,13^, and the RAD score^7,14^. “Supplementary Methods S1” summarizes the details of the high-performance predictors. Multivariate logistic regression was utilized to combine those individual predictors, develop a more robust prediction model for the ovarian tumor malignancy, and also construct the radiomics nomogram^15^.

A calibration curve was used for performance evaluation, and the model fitness was examined using the Hosmer Lemeshow test^16^. The nomogram-based diagnosis performance was assessed using the receiver operating characteristics (ROC). The probabilistic malignancy score for ovarian tumors was determined using the nomogram method, and all involved patients were assigned based on the ROC curve cut-off value to low- or high-probability groups. The clinical significance of the nomograms was assessed on the cases of patients with ovarian tumors of different degrees of malignancy. We performed decision curve analysis (DCA) to check the feasibility of the nomograms^17^.

### Statistical analysis

The data normality was verified using the Kolmogorov–Smirnov test. This test was carried out using the SPSS 23.0 software and the R statistical tools (Version 3.4.4). The probability scores from the benign and malignant samples were statistically compared based on the t-test (for normally distributed data) and the Mann–Whitney U test (for data with skewed distributions), where the scores were expressed by mean ± standard deviation (x ± s). The χ^2^ test was employed in comparing count data among the two groups. The model predictive efficacy was assessed using typical diagnostic indicators such as the accuracy, the sensitivity, the specificity, as well as the area under the ROC curve (AUC)^18^.

### Ethical approval

All procedures performed in studies involving human participants were in accordance with the ethical standards of the institutional and/or national research committee and with the 1964 Helsinki declaration and its later amendments or comparable ethical standards. Officially approved and accepted by the Ethics Committee of Jiangxi Provincial People’s Hospital Affiliated to Nanchang University.

### Informed consent

Informed consent was obtained from all individual participants included in the study.

## Results

### Patient characteristics

As indicated in Table 1, no significant differences could be detected among patients from the training and test subsets with respect to the factors of age, ascites, boundary, or biomarker expression (i.e., CA125). Nevertheless, for patients with benign or malignant tumors, significant statistical differences were realized in age, ascites, CA125, and the radiomic signature (all p < 0.05). The other differences turned to be insignificant, as demonstrated in Table 2.

**Table 1:** Demographic characteristics in the training and validation sets.

	Training set (n = 95)		P-value	Validation set (n = 39)		P-value
	Benign	Malignant		Benign	Malignant	
Number	44	51		18	21	
**Age**	41.6 ± 19.0	53.4 ± 11.8	0.001	41.3 ± 17.9	52.8 ± 8.2	0.031
< 18	4(9.1%)	1 (2.0%)		1 (5.6%)	0 (0.0%)	
> 18, ≤ 30	11(25.0%)	2 (3.9%)		4 (22.2%)	0 (0.0%)	
> 30, ≤ 50	16(36.4%)	16(31.4%)		9 (50.0%)	9 (42.9%)	
> 50	13(29.5%)	32(62.7%)		4 (22.2%)	12 (57.1%)	
**CA125**			< 0.0001			0.0007
< 35	21 (47.7%)	5 (9.8%)		9 (50.0%)	3 (14.3%)	
> 35, ≤ 200	20 (45.5%)	13 (25.5%)		9 (50.0%)	5 (23.8%)	
> 200, ≤ 500	2 (4.5%)	11 (21.6%)		0 (0.0%)	3 (14.3%)	
> 500	1 (2.3%)	22 (43.1%)		0 (0.0%)	10 (47.6%)	
**Ascites**			< 0.0001			0.0002
None	30 (68.2%)	8 (15.7%)		10 (55.6%)	3 (14.3%)	
Little	11 (25.0%)	12 (23.5%)		8 (44.4%)	4 (19.0%)	
Middle	1 (2.3%)	12 (23.5%)		0 (0.0%)	5 (23.8%)	
Large	2 (4.5%)	19 (37.3%)		0 (0.0%)	9 (42.9%)	
**Boundary**			0.0003			0.024
Clear	38 (86.4%)	26 (51.0%)		17 (94.4%)	12 (57.1%)	
Intervenient	6 (13.6%)	14 (27.5%)		1 (5.6%)	4 (19.0%)	
Obscure	0 (0.0%)	11 (21.6%)		0 (0.0%)	5 (23.8%)	
Radscore median [iqr]	− 1.3 [− 2.8, 0.2]	1.6 [0.7, 2.1]	< 0.0001	− 1.5 [− 3.8, 0.3]	1.6 [1.2, 2.2]	< 0.0001

**Table 2:** Results of univariate and multivariate logistic regression for predicting malignancy in ovarian masses.

Variable	Univariate regression			Multivariate regression		
	Odds ratio	(95% CI)	P-value	Odds ratio	(95% CI)	P-value
Age	2.798	[1.577;4.963]	4.35E − 04	3.33	[1.45;7.64]	0.005
CA125	4.670	[2.508;8.695]	1.18E − 06	3.29	[1.56;6.96]	0.002
Boundary	4.947	[2.030;12.056]	4.35E − 04			
Ascites	3.970	[2.272;6.936]	1.27E − 06	2.75	[1.50;5.06]	0.001

### Performance outcomes for the clinical prediction model

The constructed clinical prediction model for identifying benign and malignant ovarian neoplasms returned the following performance metrics. For the training set, the AUC was 0.82 (with a 95% CI 0.73–0.91), while the sensitivity, specificity, and accuracy were 76.5%, 88.6%, and 82.1%, respectively. For the validation data, the AUC was 0.82 (with a 95% CI 0.68–0.96), while the sensitivity, specificity, and accuracy rates were 71.4%, 88.9%, and 79.5%, respectively (See Table 3).

**Table 3:** Predictive performance outcomes of the radiomic nomogram, radiomic algorithm, and clinical model.

Group	Model	Accuracy	95% CI	Sensitivity	Specificity
Training	Clinical	0.821	[0.729;0.892]	0.765	0.886
	Radiomics	0.811	[0.717;0.884]	0.784	0.841
	Nomogram	0.905	[0.828;0.956]	0.902	0.909
Validation	Clinical	0.795	[0.635;0.907]	0.714	0.889
	Radiomics	0.821	[0.665;0.925]	0.857	0.778
	Nomogram	0.897	[0.758;0.971]	0.947	0.850
External validation	Clinical	0.760	[0.549;0.906]	0.583	0.923
	Radiomics	0.760	[0.549;0.906]	1.000	0.538
	Nomogram	0.880	[0.688;0.975]	0.846	0.917

### Construction and assessment of the radiomic signature

**Figure 2:**
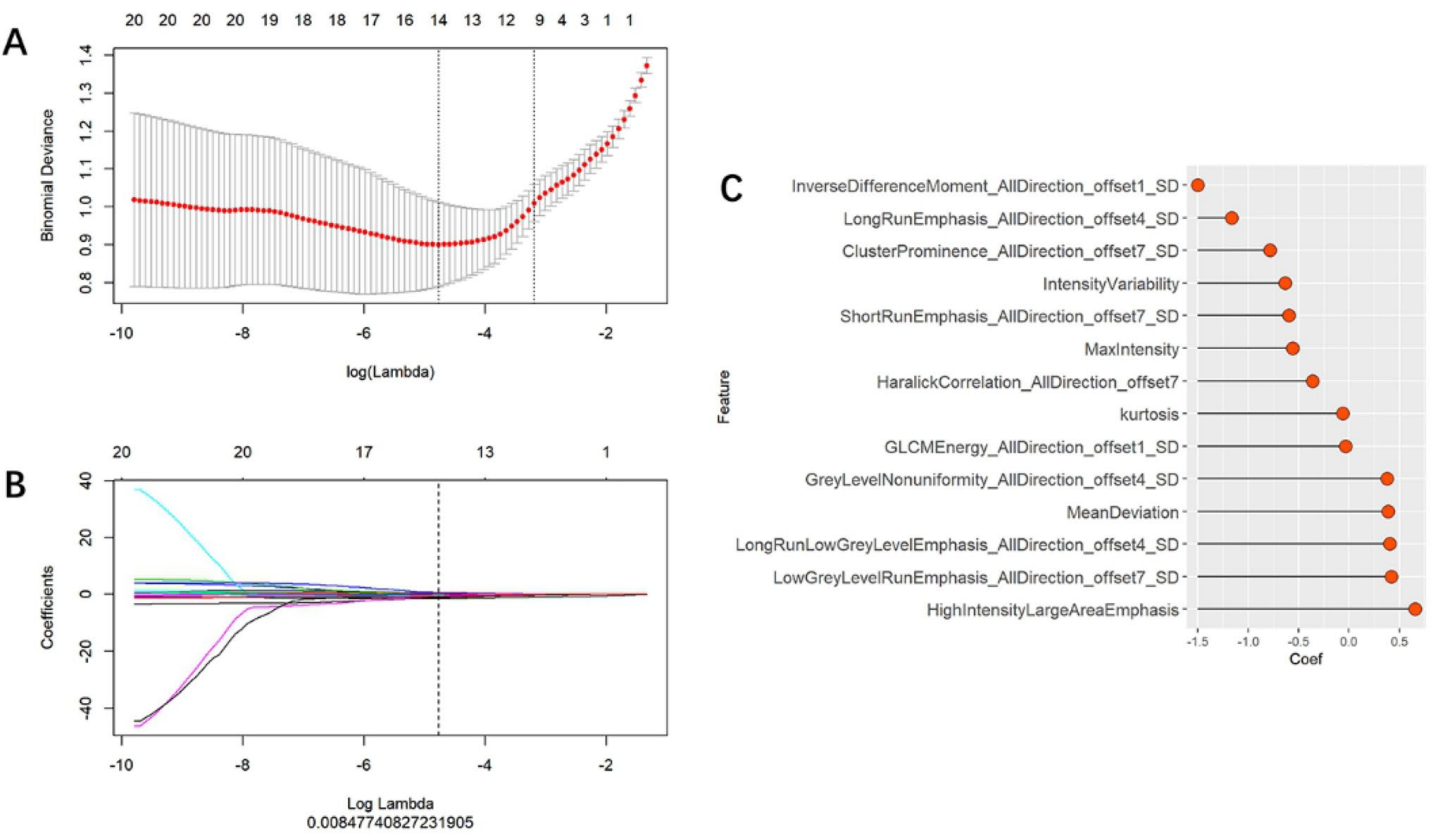
Feature selection using the LASSO-based logistic regression. (**A**) Selection of the tuning parameter (λ) using tenfold cross-validation and the minimum criteria. A plot of the partial likelihood deviance was made against log (λ). The minimum and 1-SE criteria were used to draw the dotted vertical lines at the optimal values. (**B**) Profiles of the LASSO coefficients for the 20 texture features. The vertical line was drawn at a value selected from the log (λ) sequence using tenfold cross-validation. Six features of non-zero coefficients are shown. (**C**) The selected radiomic features and corresponding coefficients.

**Figure 3:**
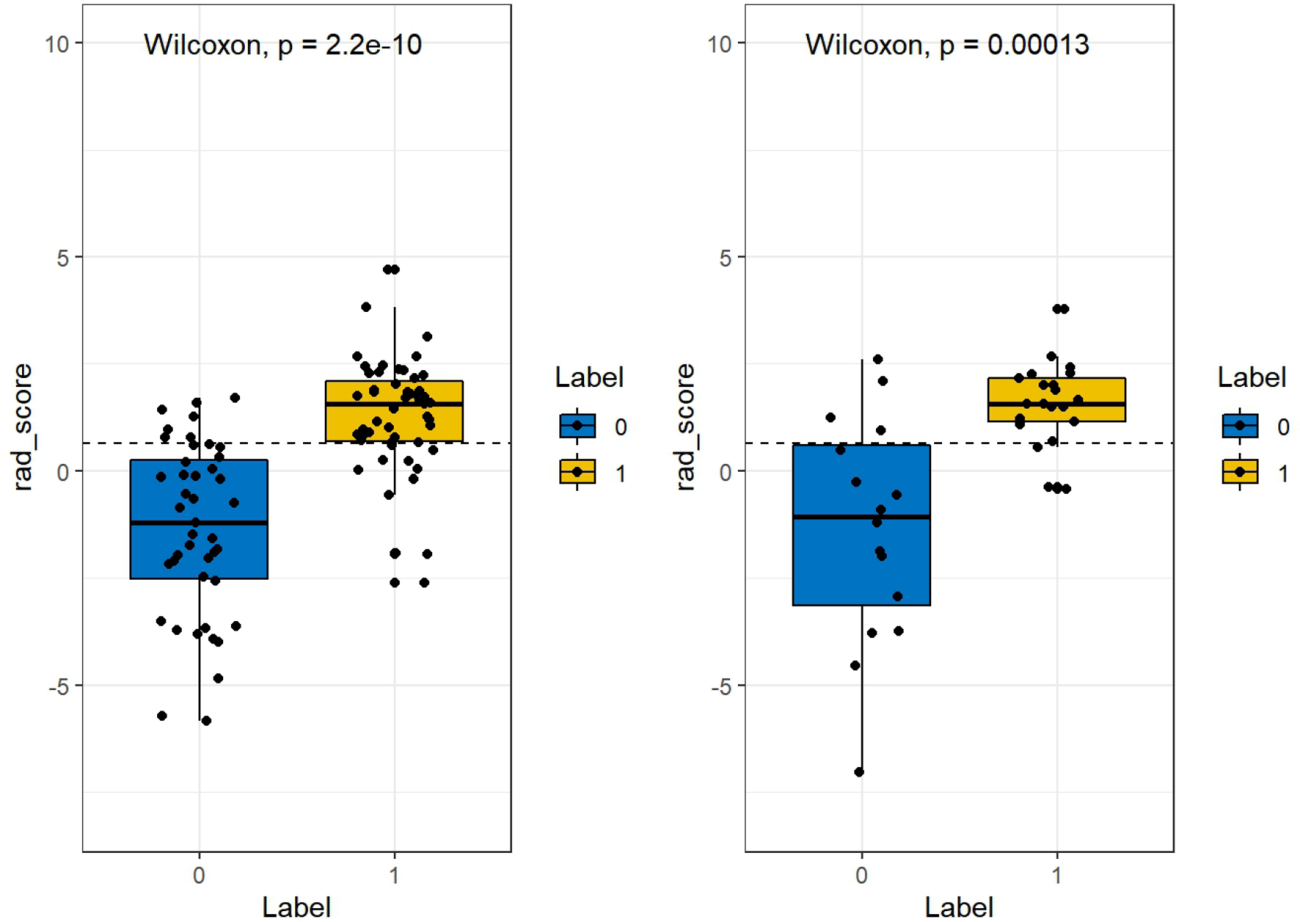
Comparison of the radscore for benign and malignant ovarian tumors on the training and test sets, respectively. (left: training set; right: test set).

Multivariate logistic regression were employed for the construction of the radiomic signature. After feature selection, 14 features were selected, which were utilized in forming the radiomic signature model (radiomics) (see Fig. 2). Our results show that good prediction performance using the radiomic signatures for both the training and test sets, with a marginal difference in performance on the two sets. Specifically, the radiomic signature exhibited favorable performance with AUC values of 0.88, 0.87 and 0.83 on the three sets, accuracy values of 81.1,82.1 and 76.0%, specificity values of 84.0, 77.7, and 53.8%, and sensitivity values of 78.4, 85.7, and 100.0%, respectively (see Table 3). The radscores showed a significant statistical difference among the benign and malignant samples for both training and testing. This indicates that the radiomic signature correlates well with the differential tumor diagnosis, as shown in Fig. 3.

### Construction and assessment of the radiomic nomogram

**Figure 4:**
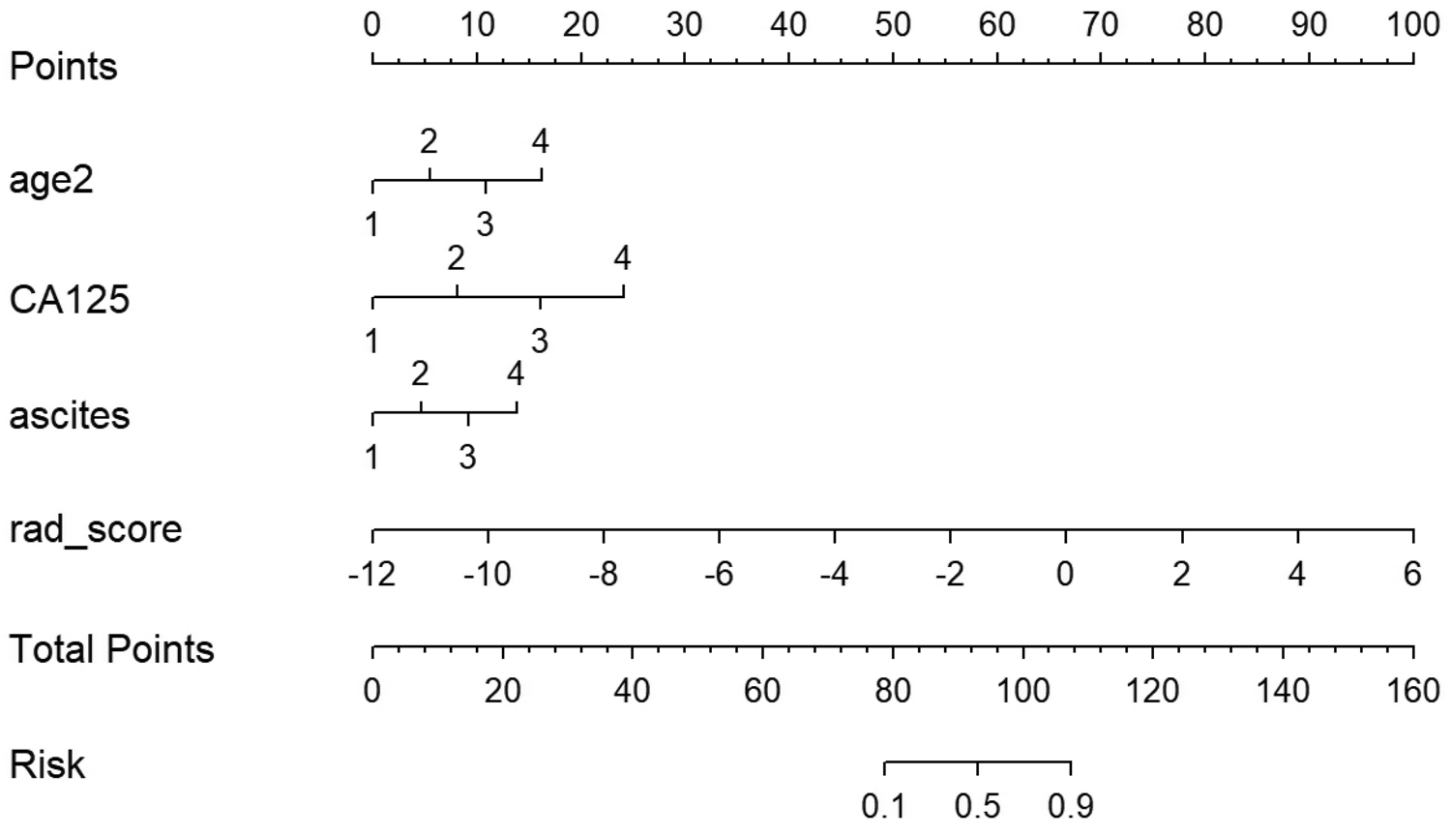
A nomogram for identifying benign and malignant ovarian tumors.

We revealed through univariate logistic regression that age, ascites, CA125, and the radiomic signature could independently predict and diagnose ovarian tumors. As shown in Table 2 and Fig. 4, multiple logistic regression was carried out using these predictors in order to construct more robust prediction models and the nomogram.

**Figure 5:**
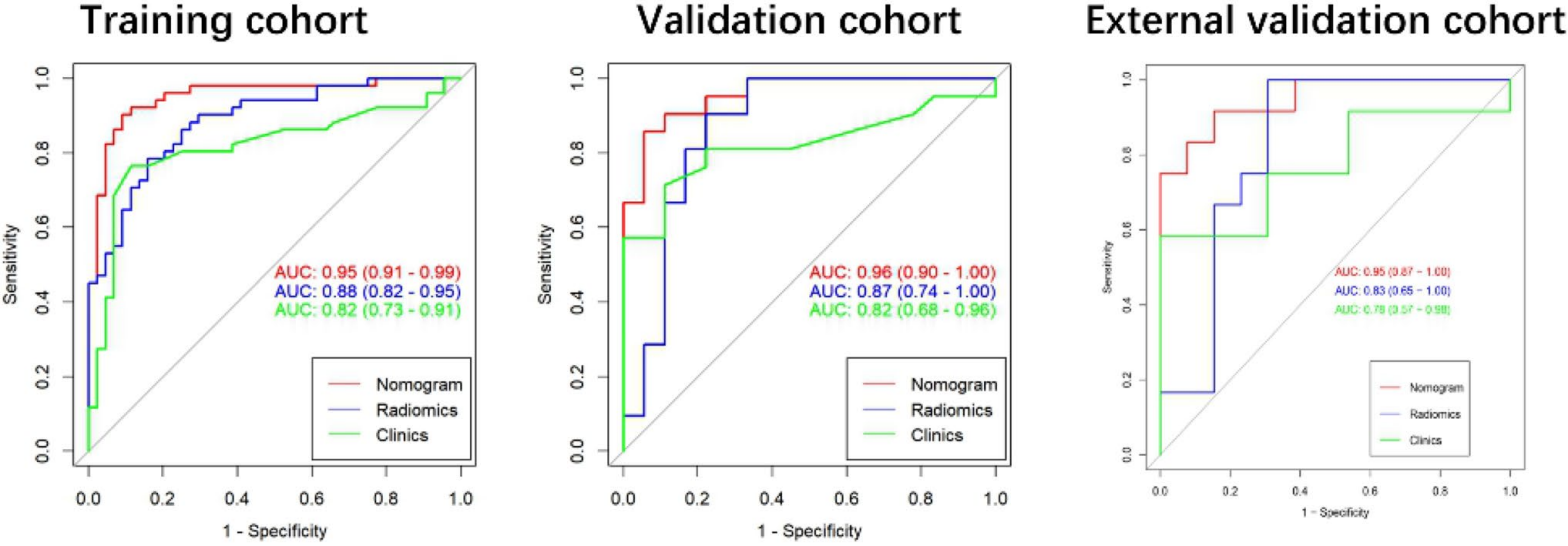
The AUC values for radiomic signatures used in identifying benign and malignant ovarian tumors.(left: training set; middle: test set; right: external set).

**Figure 6:**
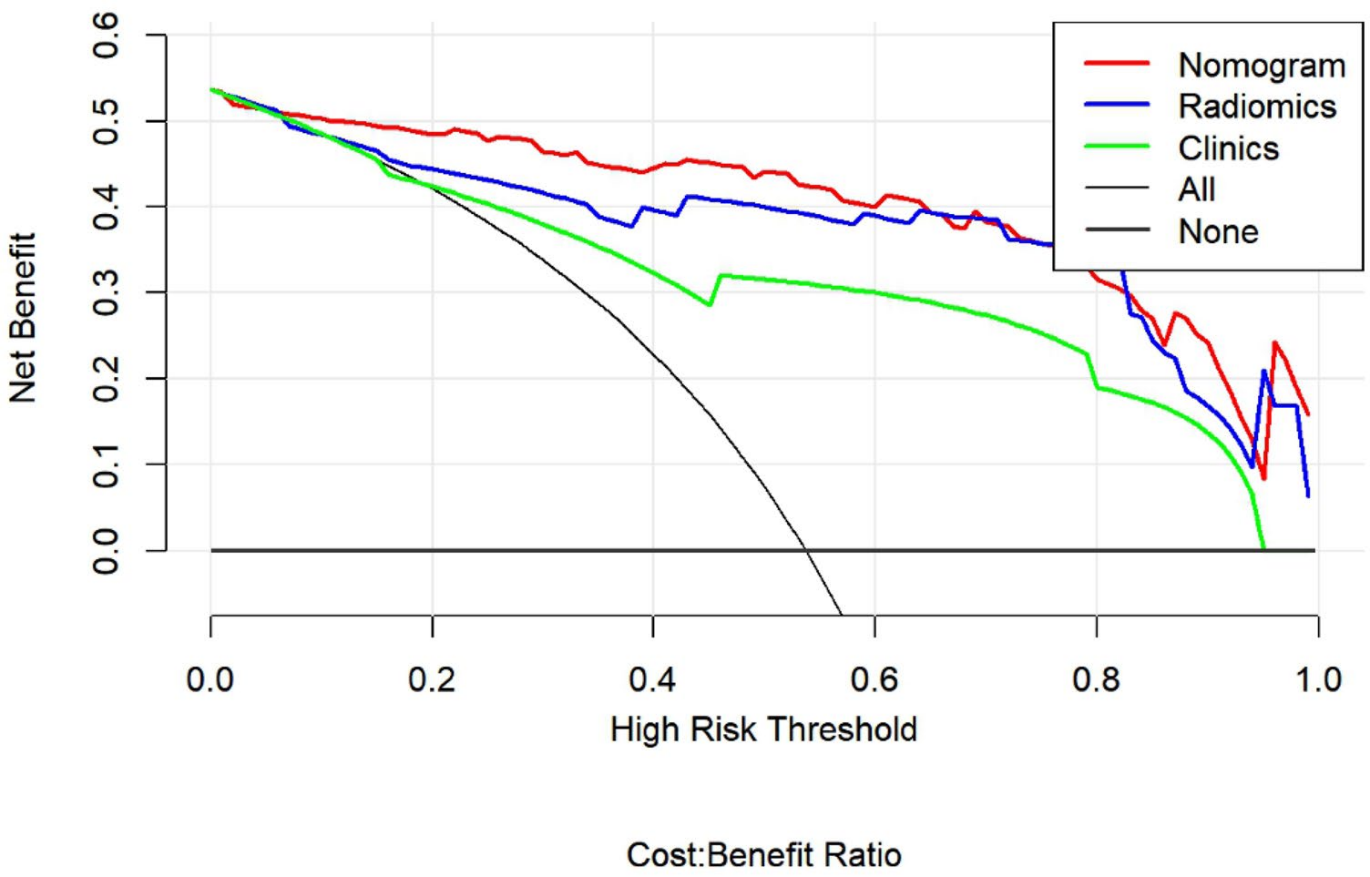
Decision curve analysis of imaging and clinicopathological features. The green, blue and red lines correspond to the nomograms from the clinical, radiomic, and nomogram models, respectively. Also, the light gray line is associated with the hypothesis that all imaging and clinicopathological features are related to ovarian malignant tumors. As well, the dark gray line is associated with the hypothesis that all imaging and clinicopathological features are not related to ovarian malignant tumors.

Excellent consistency among the predicted and actual ovarian tumor types was shown using the calibration curves in the radiomic nomograms for both patient sets. The AUC values of the nomogram-based tumor prediction in the three sets were respectively 0.95, 0.96 and 0.95. The accuracy, specificity, and sensitivity were 90.5, 90.2, and 90.9% for the training set; 89.7, 94.7, and 85.0% for the test set and 88.0, 84.6, and91.7% for the external validation set, respectively (Table 3 and Fig. 5). According to the DeLong test, the AUCs of the models based on clinical information were significantly different from the nomogram-based ones for the training and testing sets (See Table 4). Hence, the nomogram method was found to have good performance on both sets. In addition, the Hosmer–Lemeshow test demonstrated no statistically significant differences among the training and testing subsets (p > 0.05). This verifies the nomogram diagnostic superiority. The nomogram was also used to estimate the probability scores of the ovarian tumors, where patients were categorized into the low- and high-probability groups based on the Youden index^19^ (with a cut-off value of 0.391), which was defined according to the training-set nomogram. The high- and low-probability groups had a significant difference in the number of benign and malignant samples (p < 0.0001). Figure 6 depicts the DCA plot of the radiomic nomogram. Clearly, the plot shows that the radiomic nomogram method outperforms the clinical model for the “treat none” vs. “treat all” strategies with a treatment probability threshold ranging from 0 to 0.9.

**Table 4:** Comparison of the prediction with the radiomic nomogram, radiomics algorithm, and the clinical model.

Group	Model 1	Model 2	P-value
Training	Clinical	Radiomics	0.224
	Radiomics	Nomogram	0.013
	Nomogram	Clinical	0.002
Validation	Clinical	Radiomics	0.560
	Radiomics	Nomogram	0.087
	Nomogram	Clinical	0.040

## Discussion

Since the introduction of radiomics in 2012, this paradigm has been widely used in investigating ovarian tumors. Zhang et al.^7^ report that MRI-based radiomic features show high correlation with ovarian endometrioid carcinoma (OEC) classification and patient prognosis. Also, Park et al.^8^ showed that models of machine learning (ML) using age and texture features of contrast-enhanced CT resulted in high sensitivity as well as moderate specificity for malignant lesion detection^20^. However, there is currently no single CT-based texture feature to identify benign and malignant ovarian tumors. Hence, this study explored CT texture features based on plain CT scans, which has a wide range of clinical applicability.

Among the examined features, the gray-level size-zone matrix features have the largest value among the 14 features. This reflects the feature strength heterogeneity and emphasizes the extensive heterogeneity in ovarian tumors^21,22^. The form-factor features describe the 3D size and shape of the tumor area^23^. In this study, no form-factor features were consistent with the research focus on 2D plain CT images. This also indicates no statistically significant correlation between the tumor type and size.

We now consider the gray-level co-occurrence matrix (GLCM) features, that describe the frequencies of the pairwise arrangements of voxels associated with the same gray-level value. The investigated features in this study included three types of the GLCM features (namely, the energy, the inverse difference moment, and the Haralick correlation), which further characterize the heterogeneity of local tumor regions^22,24^. The grey-level run-length matrix (RLM) features reflect the texture roughness and directionality, since the value of the long-run emphasis is dominant in a rough image^25^. We found that the screening results contained 5 RLM features. In fact, three RLM features were present in the front row: low-run grey-level emphasis, long-run grey-level emphasis, and grey-level nonuniformity.

Texture analysis of CT imaging data has demonstrated promising results on various types of tumors for pathological feature prediction, prognosis, and response to therapy^6^. Meng et al.^14^ suggested that the approach of CT-based radiomics has a clear potential for differentiating between the sarcomatous renal cell carcinoma (SRCC) and the clear cell renal cell carcinoma (CCRCC). Dong et al.^15^ used a deep-learning approach in order to construct a robust predictive model based on preoperative CT images, tumor histology, and cancer grading in patients with cervical cancer. A reasonable accuracy was achieved by this model in predicting the lymph node state in cases of cervical cancer. In our work, we have built a 2D CT-based radiomic nomogram model for identifying benign and malignant ovarian tumors. The nomogram method resulted in AUC scores of 0.95 and 0.96 for the training and test sets, respectively. The nomogram method was indeed capable of providing good calibration and differentiation of ovarian tumors, and proved to be a reliable and effective method for screening malignant ovarian lesions.

In our work, we chose 2D CT-based texture signatures for the analysis of ovarian tumors. As 2D ROIs were easy to manipulate, and the proposed signatures offered lower complexity and faster computations, the use of 2D features in clinical practice is highly recommended^26^. The outcomes based on the introduced signatures for identifying benign and malignant ovarian tumors were surprisingly superior. All ovarian lesions initially emerge as small tumors that show temporal steady growth. So, the tumor volume estimate depends clearly on the imaging time. Therefore, a small or intermediate tumor volume could not be a reliable biomarker^27^. Future studies should be implemented with large expanded datasets and more clinical features. Such enhancements shall reduce the dependence of the radiomic model on relevant clinical features^4,28^.

There are several limitations of this study. First, ROI segmentation for the ovarian tumors was carried out manually. This inherently resulted in both inter-observer and intra-observer variabilities, as it has been usually the case for other cancer types. The applicability of the developed signature is limited to scans acquired with the same scanner and scanning parameters. The reproducibility of radiomic features across different acquisition and reconstruction parameters based on data with reference values (Phantom scans) will be performed in our future study to obtain a generalizable radiomic model. Second, due to the retrospective nature of the analysis, the reproducibility and comparability of the results would be hindered by potential selection bias. Third, borderline tumors were not included in this research, and this deliberate choice may cause bias. Last but not the least, besides expanding the sample set, state-of-the-art techniques (e.g. fully-automated image segmentation, feature dimensionality reduction, deep learning, and multiobjective optimization) could be further exploited for boosting classification performance.

## Supplementary Information

Supplementary Information.
