# Supplementary material for: A radiomics approach for automated diagnosis of ovarian neoplasm malignancy in computed tomography
Source: Sci Rep. 2021 Apr 22;11:8730. doi: 10.1038/s41598-021-87775-x (PMC8062553; doi:10.1038/s41598-021-87775-x)
Supplement: Supplementary file 1 — Supplementary Information. [file 41598_2021_87775_MOESM1_ESM.pdf]

Supplemental Methods

Radiomics features

396 imaging features calculated for each patient from every MRI sequence using by AK software included: (1)42 first-order intensity statistics features; (2)9 shape and size based features; (3) 154 gray level co-occurrence matrix(GLCM) features; (4) 180 gray level run-length matrix (RLM) features and (5) 11 grey-level size-zone matrix(GLZSM) features. GLCM and RLM in 4 directions (0°,45°,90°,135°) and 3 displacements (1,4,7) were calculated to describe patterns or the spatial distribution of voxel intensities. All above features were described in the study of Zhenyu Shu, et al [1].

Formula and state of Selected features

- gray-level histogram: Histogram parameters are concerned with properties of individual pixels. They describe the distribution of voxel intensities within the image through commonly used and basic metrics. Let X denote the three dimensional image matrix with N voxels and P the first order histogram divided by N discrete intensity levels. The selected following 3 first order statistics were extracted:

1) MaxIntensity: The maximum intensity value of X.
2) MeanDeviation: The mean of the absolute deviations of all voxel intensities around the mean intensity value.
3) ClusterProminence: Cluster Prominence is a measure of asymmetry of a given distribution, high values of this feature indicate that the symmetry of the image is low, in medical imaging low values of cluster prominence represent a smaller peak for the image grey level value and usually the grey level difference between the forms is small $\sum_{i,j}((i-\mu)+(j-\mu))^4 g(i,j)$

- **Form factor:** These group of features includes descriptors of the three-dimensional size and shape of the tumor region. Let in the following definitions V denote the volume and A the surface area of the volume of interest. 4 selected form factor parameters were determined as follows:

1) VolumeMM: the volume of the tumor is determined by counting the number of pixels in the tumor region and multiplying this value by the voxel size. VolumeMM denotes the volume in cubic millimeter.
2) SurfaceArea: The surface area is calculated by triangulation (i.e. dividing the surface into connected triangles) and is defined as: $A = \sum_{i=1}^{N} \frac{1}{2} |a_i b_i \times a_i c_i|$
3) Sphericity: $Sphericity = \frac{\pi^{\frac{1}{3}} (6V)^{\frac{2}{3}}}{A}$
4) Compactness2: $compactness2 = 36\pi \frac{V^2}{A^3}$

- GLCM features: The Grey level co-occurrence matrix (GLCM) **P** (i,j|**θ**,**d**) represents the joint probability of certain sets of pixels having certain grey-level values. It calculates how many times a pixel with grey-level i occurs jointly with another pixel having a grey value j. By varying the displacement vector d (distance to the neighbor pixel:1,2,3…) and different angle(**θ**)(rotation angle of an offset: 0°， 45°, 90°,135°) between each pair of pixels. 2 selected GLCM parameters were determined as follows:
  1) Entropy: a measure of randomness of intensity image. Entropy shows the amount of information of the image that is needed for the image compression. Entropy measures the loss of information or message in a transmitted signal and also measures the image information.

  $$Entropy = -\sum_{i,j} g(i,j) \log_2(i,j)$$

  2) Energy: This feature Returns the sum of squared elements in the GLCM. Range = [0 1]. Energy is 1 for a constant image. The high value when image has very good homogeneity or when pixels are very similar. The Energy is also known as uniformity, uniformity of energy, and angular second moment.

  $$energy = \sum_{i,j} g(i,j)^2$$

  g is a GLCM matrix, where i,j are the spatial coordinates of $g(i,j)$.

- RLM features: The grey level run-length matrix (RLM) **Pr**(i,j | **θ**) is defined as the numbers of runs with pixels of gray level i and run length j for a given direction θ. RLMs is generated for

each sample image segment having directions (0°,45°,90° &135°). 7 selected RLM parameters were determined as follows:

1) LongRunEmphasis: $LRE(\theta)=\frac{1}{n}\sum_{i=1}^{M}\sum_{j=1}^{N}p(i,j,\theta)j^2$

2) ShortRunEmphsis: $SRE(\theta)=\frac{1}{n}\sum_{i=1}^{M}\sum_{j=1}^{N}\frac{p(i,j,\theta)}{j^2}$

3) ShortRunLowGreyLevelEmphasis: $SRLGE(\theta)=\frac{1}{n}\sum_{i=1}^{M}\sum_{j=1}^{N}\frac{p(i,j,\theta)}{i^2 j^2}$

4) ShortRunHighGreyLevelEmphasis: $SRHGE(\theta)=\frac{1}{n}\sum_{i=1}^{M}\sum_{j=1}^{N}\frac{p(i,j,\theta)i^2}{j^2}$

5) LongRunLowGreyLevelEmphasis: $SRHGE(\theta)=\frac{1}{n}\sum_{i=1}^{M}\sum_{j=1}^{N}\frac{p(i,j,\theta)j^2}{i^2}$

6) LowGreyLevelRunEmphasis: $LGLRE(\theta)=\frac{1}{n}\sum_{i=1}^{M}\sum_{j=1}^{N}\frac{p(i,j,\theta)}{i^2}$

7) HighGreyLevelRunEmphasis: $HGLRE(\theta)=\frac{1}{n}\sum_{i=1}^{M}\sum_{j=1}^{N}p(i,j,\theta)i^2$

where n is the total number of runs.

Supplementary Reference:

1. Shu Z, Gong X, et al. MRI-based Radiomics nomogram to detect primary rectal cancer with synchronous liver metastases. Sci Rep 2019;9(1):3374
